# Editorial: The Role of Aire, microRNAs and Cell–Cell Interactions on Thymic Architecture and Induction of Tolerance

**DOI:** 10.3389/fimmu.2015.00615

**Published:** 2015-12-14

**Authors:** Geraldo Aleixo Passos, Daniella Arêas Mendes-da-Cruz, Ernna Hérida Oliveira

**Affiliations:** ^1^Molecular Immunogenetics Group, Department of Genetics, Ribeirão Preto Medical School, University of São Paulo, Ribeirão Preto, Brazil; ^2^Disciplines of Genetics and Molecular Biology, Department of Morphology, Physiology and Basic Pathology, School of Dentistry of Ribeirão Preto, University of São Paulo, Ribeirão Preto, Brazil; ^3^Laboratory on Thymus Research, Oswaldo Cruz Institute, Oswaldo Cruz Foundation, Rio de Janeiro, Brazil

**Keywords:** Thymus gland, central tolerance, Aire gene, miRNAs, cell-cell interactions, *Coxsackievirus*, exosomes, chemokines

The focus of this Research Topic is to bring new insights into central immune tolerance. To fulfill that, much has been discussed about the master in the regulation of tolerance, the autoimmune regulator (Aire) gene (1–3), the main thymus cell type that expresses this gene, and the medullary thymic epithelial cells (mTECs) (4, 5).

It includes 12 excellent contributions in the format of mini reviews or original research papers covering one or more of these aspects: promiscuous gene expression (PGE), epigenetics, miRNAs, association of the Aire gene and miRNAs, thymocyte–TEC interaction, coxsackievirus and type 1 diabetes, exosomes in the thymus, thymic crosstalk, thymic B cells, T cell development, chemokines and migration of T cells, miRNAs and the thymic atrophy, cell–cell interactions, and thymus ontogeny.

Authors raised hypothesis, discuss concepts, and show open questions. The remaining important issues to resolve questions within the central tolerance research are briefly discussed below.

The first mini review is authored by Olga Ucar and Kristin Rattay (6). They focused on the posttranscriptional control of PGE by miRNAs as well as epigenetic control involving DNA methylation, histone modifications, and topology of chromosomes. These processes represent additional factors to be explored and that might regulate the expression of Aire-independent tissue restricted antigens (TRAs), which are implicated in the central tolerance.

Are the Eph/ephrins important for thymocyte–TEC interaction? This issue was reviewed by Javier Garcia-Ceca and cols (7). The maturation of thymocytes is depending on their interaction with TECs within the thymus. Authors argue the importance of Ephs and ephrins on the intrathymic maturation of both thymic epithelial microenvironment and thymocyte maturation and on the recruitment of lymphoid progenitors into the thymus.

Another stimulating mini review is authored by Hélène Michaux and cols (8) in which they discuss the hypothesis that infection by coxsackievirus B4 (CV-B4) could be associated with etiopathogenesis of type 1 diabetes mellitus (T1D). Authors consider that besides their tropism to the pancreatic beta cells, CV-B4 could also involve the thymus. Once within this organ the virus might somehow perturbs central tolerance to the insulin family triggering thus autoimmune T1D.

Our group contributed with a mini review (9) focusing on cell–cell interactions within the thymus involving TECs and thymocytes and the role of the Aire gene on the induction of central tolerance throughout the modulation of TRA expression in mTECs. In addition, we discuss the recent evidence that Aire also regulate the expression of miRNAs in these cells. On its turn, the Aire-dependent miRNAs might exert control over TRAs. We raise issues that besides the transcriptional control exerted by Aire, PGE could also be being controlled through posttranscriptional mechanism involving miRNAs.

A very pertinent question raised by Gabriel Skogberg and cols (10) is on the role of exosomes on TRA presentation by TECs to thymocytes and its implication in the thymocyte selection. Exosomes may be liberated by TECs to the extracellular milieu and transport TRAs as well as MHC molecules, establishing intercellular communication to enhance antigen presentation to developing thymocytes. Authors discuss how intercellular communication via exosomes within the thymus could have consequences on TRA presentation and finally on central tolerance.

The thymic crosstalk, i.e., the reciprocal control by the close contact between TECs and thymocytes, which influences the differentiation of both types of cells was elegantly reviewed by Noëlla Lopes and cols (11). Authors discuss the role of dendritic cells (DCs) subsets in the process of deletion of autoreactive T cells and the generation of natural Tregs and raise questions how hematopoietic cells may control the organization of the thymic medulla.

Thymus is an organ composed of different cell types including TECs, DCs, macrophages among other cell types, and of course thymocytes. Recently, researchers have identified an unexpected cell type formed by B cells, which may be originated from intrathymic B lymphopoiesis or immigration from the periphery. Tomoyoshi Yamano and cols (12) contributed with a mini review discussing the role of thymic B cells expressing MHC-II, CD80, and Aire, in the crosstalk with CD4 single positive cells. Authors raise questions how these cells might play a role as antigen presenting cells in an unpredicted way within the thymus.

The regulation of T cell development is apparently well resolved; however, several unsolved questions remain. This important aspect is represented in this Research Topic through the mini review by Iris Caramalho and cols (13). Authors show new questions on the beginning of Treg lineage commitment, their spatial localization within the human thymus and their molecular components.

Cell migration within the thymus is crucial for the central tolerance. Developing thymocytes migrate throughout the thymus being exposed initially to the cortex and then to the thymic medulla were they respectively undergo positive and negative selection. Chemokines represent key regulators for thymocyte migration. Zicheng Hu and cols (14) argue the role of chemokines in the thymic cell migration and induction of central tolerance.

Thymic atrophy during senescence is widely recognized; however, poorly understood. In addition to the atrophy due to senescence, thymus involutes in response to a variety of stimuli including microbial infections. The mouse model of *Trypanosoma cruzi* infection corresponds to an adequate mouse model to access this question. Leandra Linhares-Lacerda and cols (15) show results on the role of miRNAs on regulation of chemotaxis, which contribute to a better understanding, while incites new issues, of thymic involution.

Cellularity of mTECs is pivotal for cell–cell interactions within the thymus, which is required for central tolerance. Taishin Akiyama and cols (16) argue the role of cytokines on cellularity of mTECs focusing into the molecular basis of cell–cell interactions opening perspective on the use of mathematical models for understanding these processes.

Thymus morphogenesis is a central point with many open questions. The mini review authored by Arnon Dias Jurberg and cols (17) addresses the role of the large superfamily of TGF-beta/bone morphogenetic protein ligands in the thymus morphogenesis and in T cell differentiation.

This Research Topic provides an international and updated insight into the latest developments and open questions on the cellular and molecular bases of central tolerance induction.

## Author Contributions

DM, EO and GP wrote the Editorial.

## Conflict of Interest Statement

The authors declare that the research was conducted in the absence of any commercial or financial relationships that could be construed as a potential conflict of interest.
